# Rural Prenatal Care by Nurse Practitioners: A Narrative Review

**DOI:** 10.1089/whr.2023.0011

**Published:** 2023-05-29

**Authors:** Monica Kneller, Edith Pituskin, Nicole L. Tegg, Colleen M. Norris

**Affiliations:** ^1^ Faculty of Nursing, University of Alberta, Edmonton, Canada.; ^2^ Cavarzan Chair in Mature Women's Research, Women and Children's Health Research Institute, Edmonton, Canada.; ^3^ Faculty of Medicine, School of Public Health Sciences, University of Alberta, Edmonton, Canada.

**Keywords:** prenatal care, rural, nurse practitioner, Canada, narrative review

## Abstract

**Background:**

Rural Canadian populations face many challenges due to their geographical isolation, including inaccessible and inequitable primary health care. Specifically, pregnant women are at risk of not receiving prenatal care (PNC) due to physical and social barriers. Inadequate PNC can have detrimental effects on both maternal and neonatal health outcomes. Nurse practitioners (NPs) are an essential group of alternative primary care providers who can provide specialized care, including PNC, to these underserved populations.

**Objective:**

The purpose of this narrative review was to identify existing NP-led rural PNC programs in other health care systems to support maternal and neonatal outcomes.

**Methods:**

A systematic search was performed to identify articles published between 2002 and 2022 on CINAHL (EBSCO host) and MEDLINE (OVID). Literature was excluded if (1) the context was based in urban centers; (2) the study focused on specialized obstetrical/gynecological-based care; or (3) the study was published in a language other than English. The literature was assessed and synthesized into a narrative review.

**Results:**

The initial search identified 34 potentially relevant articles. Five broad themes were identified, including (1) barriers to care; (2) mobile health clinics; (3) collaborative or tiered models of care; (4) telemedicine; and (5) NPs as essential primary care providers.

**Conclusions:**

The introduction of a collaborative NP-led approach to rural Canadian settings has the potential to address barriers to PNC and provide efficient, equitable, and inclusive health care.

## Introduction

Canadians, regardless of geographical location, are entitled to equitable public health care. Historically—and currently—rural populations have faced disparities in receiving accessible and equitable health care services due to provider unavailability and expansive geographical distances.^1^ As of 2021, 17.8% of Canadians live in rural areas,^2^ putting an alarming number of people at risk of lacking adequate health care. Rural Canadians are entitled not only to accessible primary care but also specialized care.

Women living in rural areas should have access to adequate prenatal care (PNC). Current barriers to accessing PNC include geographical location, associated travel time to appointments, and an insufficient number of available, qualified health care providers.^1^ Traveling significant distances for routine PNC may cause substantial financial constraints or transportation difficulties for rural women.^3^ Studies have found that restricted access to public transportation and long transit times played a significant role in the amount of PNC received.^4^ Furthermore, difficulty recruiting and retaining rural obstetricians was a major factor in the inability to provide PNC and intrapartum care.^5^

Poor or limited PNC has been associated with increased neonatal risk of stillbirth, preterm birth, low birth weight, and small for gestational age outcomes.^6^ Maternal health outcomes are correlated with the level of PNC received. Women who received intensive collaborative PNC showed decreased rates of gestational diabetes mellitus (GDM).^7^ Women who did not receive adequate PNC had an increased prevalence of postpartum anxiety and depressive disorders.^6^ The level of PNC was also directly correlated with the incidence and duration of breastfeeding.^6^ Thus, it is imperative that rural women have access to primary health care providers (PHCPs) who can provide adequate PNC to improve maternal and neonatal health outcomes.

Nurse practitioners (NPs) are essential PHCPs for rural populations. Roughly 14.5% of Canadians aged 12 years and older do not have a regular PHCP,^8^ with those aged 18–34 years being the least likely to have a regular PHCP.^8^ NPs are gaining traction in fulfilling PHCP roles, especially in rural and underserved populations.^9^ NP advanced practice roles can include diagnosis; ordering medications, diagnostic tests, and treatments; managing chronic disease; and evaluation of treatment plans.^10^ A study on NPs providing rural health care identified themes of NPs' passion for providing increased access to primary care and obtaining a wide variety of learning experiences and skills while working to their full scope of practice.^10^

By utilizing alternative PHCPs, rural populations can receive accessible, fair, and equitable health care. NP-led PNC programs may improve access to care for rural pregnant women and improve maternal and neonatal health. A rapid review of the literature found that NP-led models of PNC led to decreased infant mortality rates and improved management of women with GDM and health outcomes of their neonates.^11,12^

Research on this important topic is limited. Therefore, the purpose of this narrative review is to identify existing programs involving NPs providing PNC in rural settings to support maternal and neonatal health outcomes. A review of current programs, solutions, or models of care in different settings will provide a foundation for adapting or creating a PNC program to support pregnant women in rural Canada.

## Methods

A narrative review gathers, synthesizes, and then presents to readers what is known about a topic, which can help to identify any knowledge gaps within the literature.^13,14^ Our goal was to determine existing rural PNC programs that include NPs as providers; therefore, conducting a narrative review was most appropriate. A systematic approach to conducting this narrative review was followed to facilitate a comprehensible and reliable report.

The review took place from July to September 2022, with search term guidance from a professional librarian at the University of Alberta. Two databases, CINAHL (EBSCO host) and Medline (OVID), were searched to gather information. Focus keywords included “Nurse practitioner AND prenatal AND rural”. A comprehensive list of key search terms is found in Table 1.

**Table 1.** Key Search Terms for Identifying Literature

Main concepts (in combination with AND)	Keyword search terms (in combination with OR)
“Nurse Practitioner”	“Nurse practitioner^*^” (or “clinical nurse specialist” or “advanced practice nurse”)
Prenatal	Prenatal or pre-natal or antenatal or ante-natal or perinatal or prepartum or pre-partum or OB/GYN or pregnan^*^or obstetric
Rural	(rural or ruralization or ruralisation or rurality or ruralite^*^ or ruralism or rurally or nonurban^*^ or “non urban^*^” or villages or countryside or farm or ranch or farms or ranches or ranchland^*^ or farmland^*^ or farmstead^*^ or plantation^*^ or orchard or orchards or forest or “mountain medicine” or “cottage country” or “back country” or “bush country” or wilderness or outback or hinterland or “sheep station^*^” or “cattle station” or “fly in fly out” or (remote N2 (site^*^ or location^*^ or area^*^ or settlement^*^ or reserve or reserves or village or communit^*^)) or ((aeromedical^*^ or “air medical^*^”) N2 retriev^*^) or (“distan^*^ to” N1 (hospital^*^ or “medical service^*^” or “health service^*^”)) or (“distan^*^ from” N1 (hospital^*^ or “medical service^*^” or “health service^*^”))

Articles that matched keyword criteria were exported and uploaded into the systematic review management software, Covidence, with duplicates removed. The Mendeley Reference Manager was used as an organizational aid. Publication date limits were set as 2002–2022 to find recent literature that supports NP-led PNC for rural women. Literature was included if it was primary or secondary research of any research design, focused on NP-led prenatal clinics, took place within a rural setting, and discussed maternal or neonatal health outcomes.

Literature was excluded if the context was based in urban centers, the study focused on specialized obstetrical/gynecological-based care, or the study was published in a language other than English.

## Results

**FIG. 1.**
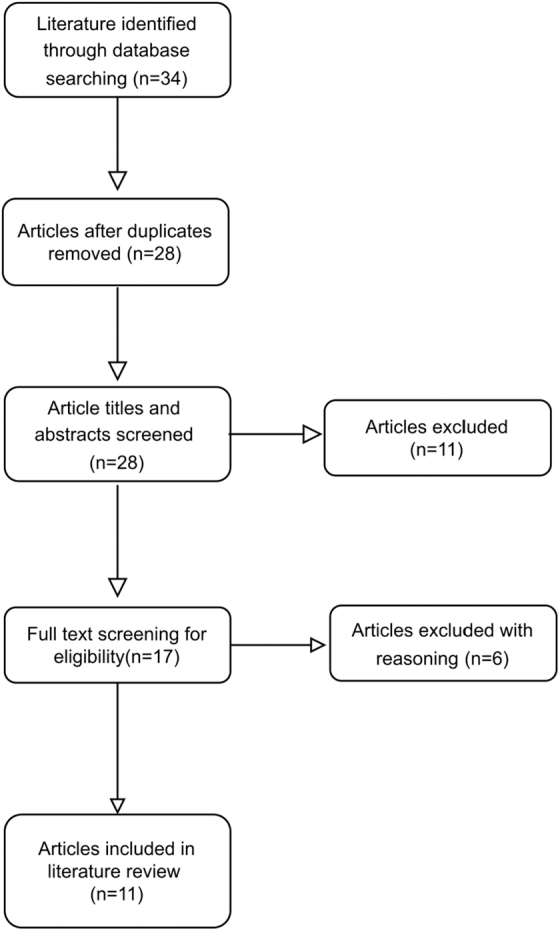
Flow diagram of literature results.

The search identified a total of 34 studies. Upon import into Covidence, duplicates were removed (*n* = 6). Title and abstract reviews were performed and studies that did not meet inclusion criteria were removed (*n* = 11). Full-text screening was executed on the remaining articles (*n* = 17), and based on appropriate inclusion and exclusion criteria, a total of 11 studies (*n* = 11) were eligible for this review. Reference lists from articles that met inclusion criteria were screened to identify additional literature. Figure 1 presents a flow diagram of the literature extracted for review. Relevant data from each source were extracted and stored on a Microsoft Excel spreadsheet (Table 2).

**Table 2.** Data Extraction from Inclusive Literature Findings

Database	Authors	Purpose	Method	Relative findings	Location
CINAHL	Bircher^21^	Describe the challenges migrant farm workers face surrounding prenatal health and access to equitable care	Editorial	Pregnant migrant workers face significant barriers to receiving prenatal care—including cost, limited clinic hours, inadequate knowledge regarding prenatal care recommendations, and migratory work with long hours and geographical isolation NPs provide safe, competent, and cost-efficient care focused on a family-centered model of care Mobile health clinic consisting of NPs bridges the gap between isolated pregnant women seeking prenatal care and primary care providers Mobile health clinics help to reduce health care costs, improve access, and promote communication between provider and patient	U.S.
CINAHL	Murfet et al.^11^	Analyze maternal and neonatal health outcomes pre- and postintervention of an NP-led model of care for managing diabetes in pregnancy	Uncontrolled interventional study Sample size: preintervention *n* = 112 and postintervention *n* = 149	Collaborative model comprising an NP, diabetes educator, obstetrician, midwife, and dietician There were no significant differences on maternal outcomes, yet substantial neonatal health benefits were observed There was a 24% reduction in adverse neonatal outcomes, including fetal macrosomia, hypoglycemia, respiratory distress syndrome, and congenital abnormalities	Australia
MEDLINE	Pinto et al.^15^	Detect challenges that obstetric providers (currently and/or previously) face in providing prenatal care and identify core components necessary for alternative methods of obstetrical care	Qualitative study with in-depth semistructured interviews and 46 health care professionals working in an obstetrical system in rural Georgia	Tiered model of risk-appropriate care based on specific needs of the prenatal population Enhances provider efficiency and streamlines patient care by having patients see an appropriate level of provider based on individual needs NPs providing prenatal care to low-risk women allows for increased availability of obstetricians to care for high-risk pregnant women Some specialists prefer to care for all levels of prenatal patients regardless of risk A clear, sustainable alternative model of care lacks consensus, thus different approaches are community specific	U.S.
CINAHL	Snyder and Thatcher^12^	Review the effects of implementing an NP model providing prenatal care in a mobile health clinic reaching out to rural populations	Article	Geographical barriers to people in Appalachia receiving medical care—specifically women Before NPs' arrival, the local county had the highest infant mortality rate, with delay of prenatal care being one of the major contributing factors “Health Wagon”—implementation of a mobile NP-led clinic providing a wide variety of health care services to rural families As a result, the county had a decrease in infant mortality rates from an average of 6.69 deaths per year to 1.86 per year	U.S.
MEDLINE	Hansen et al.^19^	Identify a potential correlation between providers' knowledge and support of transvaginal ultrasound to screen for preterm birth risk and the actual incidence of preterm birth for rural women	Mixed methods study Semistructured interviews and survey scale Sample size: 14 health care providers	Preterm birth prevention through cervical length screening *via* transvaginal ultrasound during pregnancy Rural women are at a disadvantage of receiving adequate prenatal care due to limited providers, lack of resources, multiple comorbidities, and insufficient public transportation Factors contributing to lack of cervical length surveillance include lack of access to sonography, patient noncompliance, and socioeconomic factors, including financial constraints and inability to afford transportation Low health literacy and lack of prenatal care are factors contributing to women not recognizing long-term consequences of preterm birth	U.S.
MEDLINE	Mills et al.^20^	Pilot program to advance registered nurses' roles to provide antenatal services under supervision of a general practitioner	Mixed methods research design Sample size: 11 advanced practice RNs and 4 GPs	Rural nurses with advanced antenatal training provided care for low-risk pregnant women in response to the maternity service provider shortage The study has multiple limitations, but the overall conclusion is that nurses with advanced practice were able to sufficiently provide antenatal services to low-risk women living in remote areas of Australia	Australia
CINAHL	Veith et al.^17^	Pilot program to evaluate effect of collaborative prenatal care for high-risk obstetrics in rural populations with the use of advanced practice nurses and telemedicine.	Quantitative research design Sample size: 374 Comparison group: 181	NPs on site providing hands-on care, while a perinatologist attends virtually *via* telemedicine Success of the pilot program was measured in improved rates of appointment attendance and decreased adverse effects of pregnancy such as average time for neonatal intensive care unit admission Cumulative travel distance saved for patients in this 3-year study was an impressive 162,126 miles!	U.S.
CINAHL	Bruyere^22^	Editorial that argues the importance of utilizing allied health professionals such as NPs for prenatal counseling and education in response to widespread physician shortage	Editorial	A collaborative care model between physicians and NPs sharing prenatal patients improves access to consistent prenatal care and thus pregnancy outcomes NPs have longer appointment times, allowing for patients to ask questions and providing ongoing prenatal counseling and support Patients are more likely to receive earlier initial pregnancy screening, prenatal care, and education regarding lifestyle risk factors (abstinence from alcohol, smoking cessation, and healthy weight gain), resulting in a decrease in risk of preterm delivery NPs provide consistent prenatal care essential to the success of therapeutic relationships between the care provider and patient With their nursing background and values, NPs have an enhanced ability to create strong patient–provider relationships and ease patient anxiety about labor and delivery by providing support and education Continuity of care is maintained by frequent prenatal appointments that NPs can provide in collaboration with the physician at the first and last prenatal appointments Having a team of providers focusing on individualized patient-centered care provides an opportunity for the best possible pregnancy outcomes	U.S.
CINAHL	Pflugeisen et al.^16^	Compare traditional prenatal care where patients receive routine face-to-face appointments with a physician and “Virtual Visit” prenatal care where patients receive a combination of in-person and virtual visits through videoconferencing between a physician and NP	Quality improvement study Quantitative research design Sample size: 1058 women	“Virtual Visit” prenatal program developed and implemented to provide women with an alternative method of accessible care Women who had more than one child at home were interested in prenatal care that was convenient, efficient, flexible, and individualized—all highlights of the “Virtual Visit” program Offered to women in the U.S. for low-risk pregnancies Results showed that there were no increased risks to mother or baby with virtual prenatal care compared with traditional care. There was a higher incidence of women in the “Virtual Visit” program who were partnered, higher income, and had given birth before	U.S.
CINAHL	Edgerley et al.^18^	Evaluate the birth outcomes for rural underserved women receiving early prenatal care through the use of a mobile health van compared with women receiving traditional prenatal care in community clinics	Retrospective cohort study Sample size: 108 women	Mobile health van staffed with an obstetrician–gynecologist and family NP Variety of services are offered, including, but not limited to, pregnancy testing, pelvic ultrasound, pregnancy counseling, and education Initial care is conducted in the van, and the mobile clinic acts as a bridge connecting pregnant patients to local primary care clinics for further prenatal appointments On average, women who utilized the mobile van initiated prenatal care 3 weeks earlier than those who initiated prenatal care at their local clinic Women (79.6%) who received care through the mobile van did so in the first trimester of pregnancy, in comparison with 59.8% of nonvan patients	U.S.

NP, nurse practitioner; U.S., United States.

A variety of research designs were used in the identified literature, including qualitative studies,^15^ quantitative studies,^16,17^ retrospective cohort design,^18^ uncontrolled interventional studies,^11^ and mixed methods studies.^19,20^ Two editorials and an article also met inclusion criteria and thus were utilized to support evidence found in the other studies.^12,21,22^ Participants included health care providers and/or pregnant women. No specific age range was noted for the pregnant participants. Sample sizes varied across studies, with participant numbers between 14 (PHCPs) and 1058 (pregnant women). Common themes identified from a review of the literature are listed in Table 3.

**Table 3.** Common Themes Identified from a Review of the Literature

Theme	Categories	Sources
Barriers to care	Rural population that is geographically isolated Lack of public transportation Financial constraints with traveling for equitable care	^12,17–19,21^
Mobile health clinics	Mobile prenatal care improving equity and access Reduction in infant mortality rates Improved neonatal outcomes	^12,18,21^
Collaborative or tiered models of care	Shared care involving physicians, NPs, obstetricians and gynecologists, perinatologists, and other specialists (as required) Tiered model to promote each level of provider to work to their full scope of practice	^11,15,17,22^
Telemedicine	Provide/receive medical care through telephone or videoconference Cost-efficient and convenient for rural populations “Virtual Visit” prenatal program	^16,17^
NP as an essential primary care provider	Longer appointment times Nursing background and values incorporated into care Promote provider–patient therapeutic relationship Consistent care	^19,20,22^

### Barriers to care associated with geographic location

A recurring theme identified in the literature was barriers to care, especially geographical location of women and the associated likelihood of not accessing early, adequate, and consistent PNC.^12,17–19,21^ There is a lack of public transportation in most rural areas, with women being required to travel significant distances *via* their own means of transport for PNC. Women with low-risk pregnancies commonly attend ∼14–16 prenatal appointments.^16^ If the closest available PNC provider is hundreds of kilometers away in both directions, finding reliable transportation and the associated financial burden may significantly affect attendance at PNC.

### Delivering PNC through mobile health clinics

Mobile health clinics have consistently demonstrated improved earlier and consistent access to PNC.^12,18,21^ A retrospective cohort study following the implementation of a community mobile van in a rural underserved population reported that women who utilized the van received initial prenatal screening and care ∼3 weeks earlier than those who went to community clinics.^18^ Seventy-nine percent (79.6%) of the women who received care through the mobile van did so in their first trimester of pregnancy, in comparison with 59.8% of their nonvan counterparts.^18^

Another study assessing the neonatal benefits of mobile health clinics in rural areas found that the infant mortality rate dropped from an average of 6.69 deaths per year to 1.86 per year.^12^ While the individual benefit of maternal outcomes is not clearly identified, the studies report significant improvements in neonatal health outcomes following implementation of mobile health clinics.

### Collaborative or tiered models of care

Collaborative or tiered models of care—where women are cared for collaboratively or by different levels of provider engagement based on pregnancy risk—including a team of physicians, NPs, and specialists as required (obstetricians and gynecologists and perinatologists), are key to providing maximal individualized PNC. Multiple studies have reported the benefits of incorporating a collaborative or tiered model of care.^11,15,17,22^ When alternative PHCPs work to their full scope of practice and care for appropriate-risk patients, specialists have more time to focus on patients who require higher risk care.^15^

Murfet et al.'s study focusing on a collaborative care model to provide PNC to rural women found a 24% reduction in adverse neonatal outcomes, including fetal macrosomia, hypoglycemia, respiratory distress syndrome, and congenital abnormalities.^11^ Thus, health care professionals working collaboratively in a shared model of care promote holistic highly personalized care, with demonstrated neonatal benefits.

### PNC provided *via* telemedicine

Telemedicine—medical care provided and received through the use of telephone or videoconferencing—was identified as a solution to promoting equitable PNC to remote populations.^16,17^ The use of telemedicine was reported to improve appointment attendance and access to care and was a cost-efficient and convenient alternative option for those who are geographically isolated. Veith et al. report that in their 3-year study, telemedicine saved their participants an astonishing 162,126 miles of travel to and from appointments.^17^

### NPs as alternative primary care providers

NPs and advanced practice nurses are essential alternatives to the widespread PHCP shortage. Multiple studies included discussions surrounding the importance of the nursing values that NPs implement into their practice to provide holistic care.^19,20,22^ NPs report longer appointment times, allowing for the development of therapeutic relationships between provider and patient, opportunities for patients and their families to ask questions, and family involvement in the pregnancy.^22^

## Discussion

Five broad common themes related to rural PNC programs were identified, including issues surrounding barriers to care; mobile health clinics; collaborative or tiered models of care; telemedicine; and NPs as essential PHCPs. Physical and social (financial) barriers (including geographical isolation; lack of public transportation; long distance and travel time required to receive adequate health care; and associated costs of travel such as time, fuel, and overnight accommodations, *etc*.) challenge the notion of equity, diversity, and inclusivity that is the basis of global health initiatives.^23^

Given the expansive geography of Canada, many rural populations simply cannot gain access to equitable health care. Identifying and acknowledging these significant obstacles are essential steps toward improving accessibility and subsequent fair, diverse, and inclusive care for all Canadians regardless of their location. Specifically, overcoming physical and social barriers is critical to rural pregnant women receiving appropriate PNC and education and it has been reported to improve maternal and neonatal health outcomes.

Other common themes identified in this narrative review suggest potential solutions to overcoming barriers and improving health care accessibility for all Canadians. Of note and relevant to the Canadian landscape were studies that reported on the implementation and outcomes of mobile health clinics specifically managed by NPs.^12,18,21^ The neonatal benefits reported were significant, including reduced rates of fetal macrosomia, hypoglycemia, respiratory distress syndrome, congenital abnormalities, and infant mortality.^11,12^

In addition to improved health outcomes, Bircher demonstrated that the use of mobile health clinics helped to reduce health care costs, improve access, and promote communication between the provider and patient.^21^ NPs delivering PNC to rural populations through mobile health clinics imply the potential for significant improvements in access, equity, consistency, and inclusivity in maternal and newborn health care.

Telemedicine through the telephone or videoconferencing between providers/specialists and patients was also a modality with potential to improve accessibility to individualized PNC. The reports indicated that this method of care breaks down the physical, social, and financial barriers faced by rural populations and could offer equitable health care to a larger number of Canadians. For example, an innovative program in the United States (U.S.) evaluated the effects of a collaborative PNC model for high-risk pregnancies in rural communities.

NPs were on site, providing hands-on care, while a perinatologist attended appointments virtually through telemedicine. The success of the pilot program was measured in terms of improved rates of appointment attendance and decreased adverse effects of pregnancy, such as the average time for neonatal intensive care unit admission.^17^ A similar program could be adapted to the rural Canadian context. This would include NPs providing face-to-face PNC, while specialists attend appointments virtually, ensuring that women who require specialized care can receive it without facing geographical, social, or financial obstacles.

A program that includes different members of the health care team in combination with the mother, baby, and family could support a collaborative holistic model of care.

A final theme identified described collaborative or tiered models of care. Importantly, Murfet et al. report that there was a significant decrease in adverse neonatal outcomes when using a collaborative care model that included NPs, general physicians, diabetic educators, and other specialists.^11^ While tangible evidence of improved maternal and neonatal outcomes is limited, NPs are essential PHCPs.

Using their full scope of practice, NPs provide accessible and equitable health care to rural populations, especially with widespread physician shortage.^20,22^ Specifically, NPs are a safe economical option for providing low-risk PNC to women living in geographically remote and/or rural settings. Furthermore, providing in-place PNC will address the most basic barriers that women face, including the time and travel and added expense required to travel to urban centers for routine prenatal appointments.

A shared-care model of care involving NPs alongside family physicians allows for consistency of care, the ability to establish a therapeutic patient–provider relationship, and adequate appointment times that will encourage prenatal education and questions that the patient or family may have.^22^

### Limitations

The limited number of available articles for review and analysis is likely indicative of the paucity of evidence related to health care provided to people in rural/remote locations. However, we were purposeful in attempting to identify studies that were completed in geographical areas similar to the Canadian landscape. While neonatal health outcomes were identified in the review, the reporting of maternal health outcomes were restricted to pilot programs.

Studies published in English only were included in this narrative review, and as a result, this may have circumscribed the available literature on NPs providing PNC. Finally, the settings for the studies in this review were limited to the U.S. and Australia. However, there is reason to suggest that the health care systems of these first world countries are comparable with Canada, therefore applying the principles and programs implemented in the U.S. and Australia may assist in adapting the evidence to the Canadian public health care system.

## Conclusions

The physical, social, and financial barriers related to the vast geography of Canada challenge the notion of equity, diversity, and inclusivity guaranteed through a publicly funded health care system. This review identified five common themes associated with providing equitable PNC for women in rural settings. NPs offer a viable option to address the identified barriers to PNC through mobile health clinics and/or telemedicine.

Furthermore, the introduction of a collaborative NP-led approach to rural Canadian settings has the potential to address barriers to PNC and provide efficient, equitable, and inclusive health care.

## Abbreviations Used

GDM: gestational diabetes mellitus
NPs: nurse practitioners
PHCPs: primary health care providers
PNC: prenatal care
U.S.: United States
